# Movement-Based Interventions in Patients Affected by Bone Metastases: Impact on Physical Function and Functional Autonomy—A Systematic Review

**DOI:** 10.3390/cancers17193266

**Published:** 2025-10-09

**Authors:** Giorgia Petrucci, Agnese Broccolo, Anna Marchetti, Chiara Monterosso, Giuseppe Casale, Chiara Timarco, Tea Zeppola, Silvia Dsoke, Elena Sandri, Michela Piredda, Giuseppe Francesco Papalia, Maria Grazia De Marinis

**Affiliations:** 1 Operative Research Unit of Orthopaedic and Trauma Surgery, Fondazione Policlinico Universitario Campus Bio-Medico, Via Alvaro del Portillo 200, 00128 Rome, Italy; g.petrucci@policlinicocampus.it (G.P.); g.papalia@policlinicocampus.it (G.F.P.); 2 Department of Biomedicine and Prevention, Tor Vergata University, Via Montpellier, 00133 Rome, Italys.dsoke@policlinicocampus.it (S.D.); 3 Research Unit Nursing in Palliative Care, Department of Medicine and Surgery, Fondazione Policlinico Universitario Campus Bio-Medico, Via Alvaro Del Portillo 200, 00128 Rome, Italy; g.casale@policlinicocampus.it (G.C.); c.timarco@policlinicocampus.it (C.T.); m.demarinis@policlinicocampus.it (M.G.D.M.); 4 Research Unit Nursing Science, Department of Medicine and Surgery, Campus Bio-Medico di Roma University, Via Alvaro Del Portillo 21, 00128 Rome, Italy; c.monterosso@policlinicoumberto1.it (C.M.); m.piredda@unicampus.it (M.P.); 5 Medical Oncology, Fondazione Policlinico Universitario Campus Bio-Medico, Via Alvaro del Portillo 200, 00128 Rome, Italy; t.zeppola@policlinicocampus.it; 6 Research Unit of Oncology, Department of Medicine and Surgery, Università Campus Bio-Medico di Roma, Via Alvaro del Portillo 21, 00128 Rome, Italy; 7 Faculty of Medicine and Health Sciences, Catholic University of Valencia San Vicente Mártir, C/Quevedo 2, 46001 Valencia, Spain; elena.sandri@ucv.es; 8 Oncological Orthopaedics Department, IFO–IRCCS Regina Elena National Cancer Institute, Via Elio Chianesi 53, 00144 Rome, Italy

**Keywords:** advancer cancer, bone metastases, movement, exercise intervention, palliative care, Activities of Daily Living, physical function

## Abstract

**Simple Summary:**

Patients with advanced cancer often develop bone metastases, which can cause pain, weakness, and loss of independence in daily life. These changes not only reduce physical function but also have a strong impact on quality of life. Staying active and exercising may help to preserve mobility, strength, and independence, but many patients and health professionals are unsure whether physical activity is safe in this context. This study brings together and summarizes the available evidence on exercise programs for people with bone metastases. The results show that structured physical activity is safe and can improve walking ability, muscle strength, energy levels, and performance in everyday tasks. These findings highlight the importance of including personalized exercise as part of supportive care, with the potential to enhance both clinical practice and future research in cancer care.

**Abstract:**

**Background:** Bone metastases are a common complication in patients with advanced cancer. These patients often experience a decline in physical function and autonomy, particularly in the ability to perform Activities of Daily Living, and structured movement-based interventions may represent an important supportive strategy. The aim of this study is to describe the available evidence regarding the impact of physical activity and exercise interventions on functional status and ADL performance in patients with bone metastases. **Methods:** A systematic literature review was conducted in PubMed, Scopus, Embase, Web of Science, and CINAHL database up to March 2025 and reported according to PRISMA guidelines. Eligible studies included adults (≥18 years) with confirmed bone metastases who underwent physical activity interventions designed to enhance functional status and ADLs. Studies’ methodological quality was assessed using the Joanna Briggs Institute critical appraisal tools, selected according to study design. **Results:** Eleven studies were included: four randomized controlled trials, four quasi-experimental studies, one randomized feasibility trial, one cross-sectional observational study, and one case report. Despite heterogeneity in intervention type, duration, and outcome measures, most studies reported improvements in physical function, including mobility, muscle strength, walking capacity, and endurance, as well as enhanced performance in ADLs and reductions in fatigue. No serious adverse events were reported. **Conclusions:** Structured physical activity appears safe and may improve function and independence in patients with bone metastases. These findings support the integration of individualized exercise programs into multidisciplinary supportive care.

## 1. Introduction

The term advanced cancer is commonly used to describe malignancies with a low probability of cure. Additionally, the term may refer to tumors that have spread from their original site to adjacent tissues, lymph nodes, or other parts of the body [1,2,3]. In clinical practice, the term advanced cancer often refers to malignancies at an advanced stage, generally not suitable for curative therapy. A significant number of patients with advanced cancer also present with metastases [4]. The localization of metastases is strongly influenced by the site of the primary tumor, although the bones represent the most common sites of dissemination [2,5]. In fact, the skeletal system is a preferential site for several solid neoplasms, including prostate, breast, and lung carcinomas [6,7,8]. In patients with advanced-stage prostate cancer, bone metastases are present at the time of diagnosis in up to 60% of cases [9]. In metastatic breast cancer, bone is the most frequently involved site (up to 64%) [9]. Similarly, in lung cancer, bone metastases are detected in almost 36% of cases [9]. The most commonly affected skeletal sites include the spine, sternum, ribs, and pelvis [9,10], followed to a lesser extent by the femur and humerus [11]. As life expectancy increases in these patients, the occurrence of skeletal metastases is also rising, with over 1.5 million individuals worldwide currently living with bone metastases [12]. Patients with bone metastases experience a wide and complex range of symptoms, which significantly impact quality of life (QoL) [13,14]. QoL is impaired by multiple factors, including pain [15,16,17], fatigue, skeletal-related events (SREs) [12], psychological symptoms such as anxiety and depression [18,19], sleep disturbances, loss of interest in activities, and neurological disorders [14]. Due to this disabling symptomatology, patients with bone metastases frequently experience a reduction in functional autonomy, particularly in Activities of Daily Living (ADLs) [20,21]. The impairment of these capabilities can lead to a deterioration in overall health status and a significant reduction in QoL [22]. In this patient population, along with the decline in QoL and ADLs, a decrease in physical functioning is often observed [23], understood as the ability to integrate motor and functional skills to perform coordinated and efficient movements necessary for daily activities, such as walking or climbing stairs [24]. The importance of independence and being able to perform ADLs autonomously is also highlighted in a descriptive study from the Netherlands [25], which found that the loss of functional autonomy is a key factor in patients’ perception of suffering when requesting euthanasia. Similarly, a Canadian qualitative survey [26] showed that the desire to maintain control and independence, especially in the face of physical decline, is a major reason for considering medical assistance in dying.

Given the chronic and often progressive nature of the disease of these patients, they are frequently included in palliative care pathways, aimed at improving QoL through a multidimensional approach [27]. Given the great impact due to the functional and psychosocial consequences associated with bone metastases, interventions aimed at preserving or improving physical function and independence have become an important goal in the care of patients with bone metastases. Among these, several structured exercise programs have been described in the literature and have shown promise in improving outcomes in these patients. In palliative care, the main goal of physical exercise is not to restore physical activity but to improve quality of life (QoL) by accomplishing goals like improving mood, preventing complications, and empowering the patient by enhancing residual capacities, which promotes a sense of control and dignity that is frequently undermined by advanced oncologic conditions [28,29].

Despite the growing interest in exercise interventions for individuals with advanced cancer and bone metastases, the literature remains fragmented with respect to key functional outcomes. Previous systematic reviews have primarily focused either on the overall effectiveness and impact on QoL [30], on the safety and feasibility of physical activity in this population [31], or have taken a narrative approach to discuss clinical considerations in exercise prescription [12]. While these reviews provide valuable insights, none have specifically synthesized the evidence on how exercise interventions affect physical function and ADLs in patients affected by bone metastases. This represents a critical gap, as physical function and ADLs are closely linked to independence, QoL, and overall prognosis [32]. Maintaining or improving functional capacity can be a key element in preserving their sense of self-mastery and supporting personal dignity [33]. This focus meets the needs and preferences expressed by patients themselves, as demonstrated in a recent qualitative meta-synthesis [34] of exercise interventions in individuals with advanced cancer. The synthesis highlights that those patients valued exercise not only for its physical benefits, such as improved strength, mobility, and symptom management, but also for its psychological and existential impact.

Therefore, the aim of this systematic review is to describe and synthesize the available evidence regarding the impact of movement-based and physical exercise interventions in patients affected by bone metastases, addressing the specific gap in physical function and ADLs.

## 2. Methods

### 2.1. Eligibility Criteria

The PIO (Population/Intervention/Outcome) method was used to establish the eligibility criteria [35]. Studies were included if they involved (P) adult patients (aged ≥ 18 years) with a confirmed diagnosis of bone metastases, regardless of the type of primary tumor. (I) Regarding interventions, eligible studies included those implementing physical activity programs, exercise training, or movement-based strategies, whether supervised or unsupervised, therapeutic or rehabilitative, aimed at improving physical function and autonomy in patients with bone metastases. (O) Studies assessing at least one outcome related to physical functioning, defined as the ability to perform daily physical tasks or ADLs.

Studies involving patients under 18 years of age (pediatric or adolescent populations) were excluded. No restrictions were applied based on sex, disease stage, or treatment status, provided that the presence of bone metastases was documented and the intervention focused on physical activity, exercise, or movement-based strategies.

### 2.2. Information Sources

This systematic review was previously registered in PROSPERO (registration number: CRD420251037984). A systematic literature search was conducted across five major electronic databases: PubMed, Scopus, Cumulative Index to Nursing and Allied Health Literature (CINAHL), Web of Science, and Excerpta Medica dataBASE (Embase). The search included all available literature up to March 2025. No restrictions were applied in terms of publication date, language, or type of publication. Studies that were not peer-reviewed, such as conference abstracts, editorials, letters, commentaries, or opinion pieces, were excluded. Additionally, secondary studies were excluded, including systematic reviews, narrative reviews, meta-analyses, scoping reviews, overviews, clinical guidelines, consensus reports, and other types of evidence syntheses.

### 2.3. Search Strategy

The search strategy was developed using a combination of controlled vocabulary (such as MeSH—Medical Subject Headings) and free-text keywords, tailored to the syntax and specific thesauri of each database. The objective was to identify studies investigating the effects of movement, mobility, or physical activity on physical functioning and ADLs in cancer patients with bone metastases.

The following search string was used as a starting point in PubMed: (“Neoplasm Metastasis” [Mesh] OR “neoplasm metastas” OR “bone metastas” OR “skeletal metastas*”) AND (mobility OR “Movement” [Mesh] OR “Locomotion” [Mesh] OR movement* OR locomotion* OR “Motor Activity” [Mesh] OR “Exercise” [Mesh] OR “motor activit*” OR exercis*) AND (“Functional Status” OR “Functional Dependence” OR “Functional Independence” OR “Activities of Daily Living” [Mesh] OR “Functional Status” [Mesh] OR “Activities Daily Living” OR “Daily Living Activit*” OR ADL OR “Chronic Limitation of Activity”). The strategy was applied consistently and adapted to the controlled vocabulary and syntaxes specific to each database (e.g., Emtree for Embase and CINAHL Headings for CINAHL), with the aim of maximizing the sensitivity and specificity of the search. The complete search strategies for each database are provided in Supplementary File S1.

### 2.4. Selection Process

The literature search was conducted up to March 2025, identifying a total of 686 studies. The screening and selection process was carried out using CADIMA, a free, web-based tool that supports the transparent planning, conduct, and reporting of systematic reviews and evidence maps. After removing duplicates, 462 studies were screened. Subsequently, two independent reviewers (A.B. and C.M.) independently assessed the titles and abstracts to determine study eligibility based on the inclusion criteria. In cases of disagreement between the two reviewers, the final decision was reached through discussion and with the involvement of a third reviewer (G.P.).

### 2.5. Data Collection Process

Once the full texts were obtained, data collection was carried out using extraction tables, one for each of the included studies. The tables were compiled to provide a comprehensive and detailed overview of the main characteristics of the selected studies. Data extraction was conducted systematically and verified by two independent reviewers (A.B. and C.M.) to ensure the accuracy and consistency of the information. Any discrepancies were resolved through direct discussion or, if necessary, with the involvement of a third reviewer (G.P.).

### 2.6. Data Items

For each of the included studies, data were extracted regarding bibliographic characteristics (author, year, title, country), study design, the population involved, and the interventions analyzed. Particular attention was given to the description of participants’ clinical conditions, with reference to cancer diagnosis, the presence of bone metastases and their stability, as well as the characteristics of the motor interventions proposed (type, intensity, duration, delivery setting). Clinical and functional outcomes were also collected, with a specific focus on the assessment of functional status and autonomy in ADLs, as reported by the authors and measured using the tools they employed. Data extraction was conducted using a predefined form, without the use of automation tools, and it was not necessary to contact the authors for additional information or clarifications.

### 2.7. Risk of Bias Assessment

The methodological quality of the included studies was assessed using the critical appraisal tools developed by the Joanna Briggs Institute (JBI), selected according to the design of each study. Two independent reviewers (A.B. and C.M.) applied the appropriate tool autonomously. Discrepancies were resolved through direct discussion or, if necessary, with the involvement of a third reviewer (G.P.). Each checklist consists of a series of questions (up to a maximum of thirteen, depending on the tool), with the possible responses being: “yes”, “no”, “unclear”, or “not applicable”. To ensure consistency in the risk of bias classification across different checklists, the percentage of “yes” responses over the applicable items was calculated for each study. The risk of bias was classified as low when the percentage of “yes” responses was equal to or greater than 80%, moderate when between 60% and 79%, and high when below 60%.

## 3. Results

### 3.1. Study Selection

**Figure 1:**
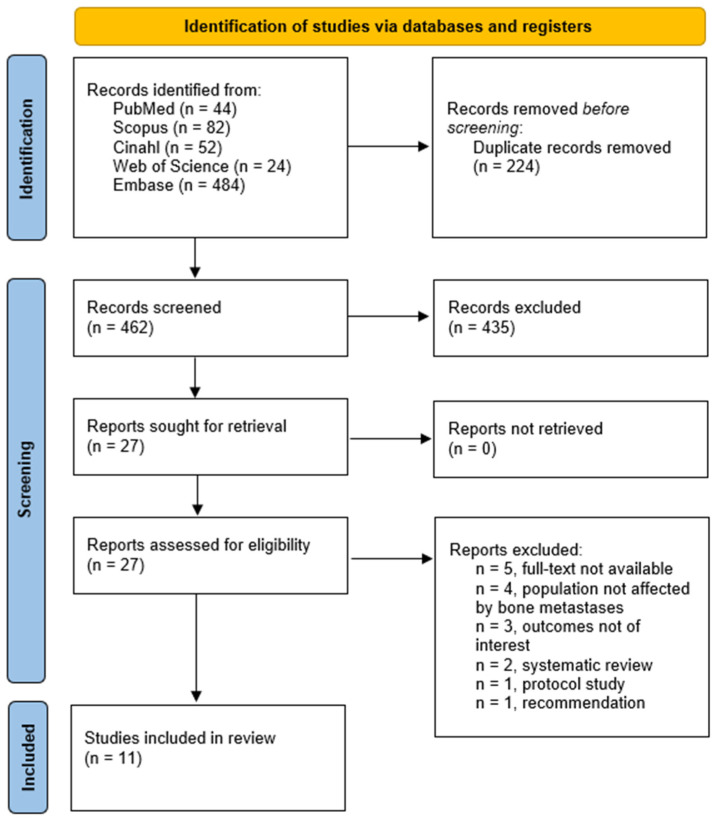
Flow diagram (PRISMA 2020) illustrating the inclusion process.

From the initial screening of 462 titles and abstracts, 438 studies were excluded as they did not meet the predefined criteria. Therefore, 24 articles were assessed in full text, of which 13 were excluded: five were conference abstracts or other non-peer-reviewed sources, four studies included populations not affected by bone metastases, three did not report outcomes relevant to physical functioning or ADLs, two were systematic reviews, one was a recommendation document, and one was a study protocol. At the end of the selection process, 11 studies were included in the systematic review. The selection process is represented in the PRISMA 2020 flow diagram [36] (Figure 1).

### 3.2. Study Characteristics

Eleven studies were included (Table 1), published between 2010 and 2024, and conducted in eight different countries: Spain, Germany, Poland, Sweden, Australia, the Netherlands, Ireland, and Japan. The methodological designs were heterogeneous. Four studies were randomized controlled trials [37,38,39,40], while four had a quasi-experimental design, including uncontrolled prospective studies or feasibility studies [41,42,43,44]. One study was a randomized feasibility trial [45], one had a cross-sectional observational design [46], and one was a case report [47].

The results of the included studies demonstrate consistent improvements in several outcomes. Among the most often improved outcomes were functional capacity and physical function. Significant improvements in muscle strength, particularly in the lower limbs, were found in several trials. Galvão et al. [40] observed an increase of +6.6 kg in lower limb muscle strength (*p* = 0.033), while Cormie et al. [42] reported a 4% increase (*p* < 0.05). Mobility and walking ability also showed significant improvement. Born et al. [47] showed an improvement of +158 m in the six-minute walk test (6MWT), while Groen et al. [43] observed an increase from 407 to 481 m. In a randomized feasibility study, Yee et al. [45] reported improvements in aerobic capacity with a VO_2_max increase of +1.6 mL/kg/min and an increase of +40 m in the 6MWT. In addition, Pajares et al. [41] highlighted a significant improvement in lower limb functional capacity, with chair rise test repetitions increasing from 14.5 to 19.61.

Several studies demonstrated a significant reduction in fatigue. Hiensch et al. [39] observed a decrease in physical fatigue of −5.3 points (95% CI: −10.0 to −0.6; *p* = 0.027). Pajares et al. [41] reported a reduction in fatigue scores from 5.54 to 4.33, while Yee et al. [45] demonstrated an improvement of +5.6 points on the FACIT-F scale. Both studies by Rief et al. [37,38] reported statistically significant reductions in fatigue (*p* = 0.013 and *p* < 0.001), and the case report by Born et al. [47] also documented a decrease from 8 to 7 points on the NRS scale.

With regard to quality of life, Hiensch et al. [39] reported an overall improvement of +4.8 points (95% CI: 2.2–7.4; *p* = 0.0003), along with enhancements in specific domains including physical functioning (+7.0; 95% CI: 3.6 to 10.3; effect size [ES] = 0.42), pain (−7.1; 95% CI: −12.1 to −1.9; ES = 0.28), dyspnea (−7.6; 95% CI: −12.2 to −3.0; ES = 0.28), and social functioning (+5.5; 95% CI: 0.2 to 10.8; ES = 0.20). Cormie et al. [42] reported a 7% improvement in social functioning (*p* < 0.05) and a 13% increase in physical role functioning at six months (*p* = 0.035). Groen et al. (2021) [43] noted a +6.2-point increase in satisfaction with daily activities and a +2.7-point improvement in overall quality of life. In the case report by Born et al. (2010) [47], social functioning improved from 25 to 50 points and vitality from 20 to 50. Finally, Yee et al. (2019) [45] reported a very large effect size (Glass’s delta = 1.71) on the physical functioning domain of the EORTC QLQ-C30.

ADLs also showed significant improvements. Abe et al. [44] found that the Barthel Index score increased from 10.0 to 40.0 (*p* < 0.001), with 92% of patients able to perform bed-to-chair transfers independently or with assistance. Groen et al. [43] observed a +6.2-point increase in satisfaction with ADLs, as measured by the USER-P scale.

Pain management also showed favorable outcomes. Hiensch et al. [39] reported a pain reduction of −7.1 points (95% CI: −12.1 to −1.9), and Rief et al. [37] observed a statistically significant decrease in pain (*p* < 0.001). Furthermore, Guinan et al. [46] found that participants who met physical activity guidelines reported lower levels of pain, with a negative correlation for worst pain (r = −0.27; *p* = 0.04).

Improvements were also observed in dyspnea, with a reduction of −7.6 points (95% CI: −12.2 to −3.0; ES = 0.28) in the study by Hiensch et al. [39], and in sleep quality, with lower insomnia scores (21.4 vs. 40.0; *p* = 0.026) among physically active participants in the study by Guinan et al [46].

In addition, the characteristics of the interventions are summarized in Table 2. The table details the type, frequency, duration, and overall length of each program. As shown, the interventions were heterogeneous, ranging from supervised multimodal exercise programs to individualized therapeutic exercise and transfer methods in palliative care. The frequency of training varied between once weekly and daily sessions, while program duration ranged from one week of habitual activity monitoring to nine months of structured supervised exercise.

In terms of safety, no significant adverse events or bone-related issues were identified in any of the included studies.

### 3.3. Risk of Bias in Studies

The quality of the studies was assessed using the JBI checklists tailored to each study design (Supplementary File S2), which included randomized controlled trials (*n* = 5), a case report (*n* = 1), a cross-sectional study (*n* = 1), and quasi-experimental studies (*n* = 4), and the overall methodological quality was found to be heterogeneous. Two studies had a low risk of bias: the case report by Born et al. [47] and the cross-sectional observational study by Guinan et al. [46]. These trials had clearly defined inclusion criteria, valid and reliable endpoints, the correct statistical tests, and extensive descriptions of the participants’ characteristics and the procedures for the intervention, often the use of standardized instruments to assess the clinical and functional benefits.

The randomized controlled trials conducted by Rief et al. [37,38], Galvão et al. [40], and Hiensch et al. [39], were classified as having a moderate risk of bias. All reported adequate randomization procedures, baseline group comparability, and consistent outcome measurements. However, several limitations were noted such as unclear allocation concealment, incomplete follow-up, and the absence of assessor blinding or intention-to-treat analyses in some cases. Five studies had a high risk of bias. This group includes the following studies: Yee et al. [45], Pajares et al. [41], Cormie et al. [42], Groen et al. [43], and Abe et al. [44].

The methodological issues common to the studies are the following: small sample size, lack of control groups, lack of baseline comparability, and limited use of blinded or intention-to-treat analyses. Among the randomized controlled trials, blinding of participants and intervention providers was not possible due to the nature of the interventions, which may have contributed to a high risk of classification bias. Overall, the level of methodological quality reflects the clinical complexity of the target population and the ethical and logistical challenges associated with conducting trials in cancer patients with bone metastases. However, the data presented, even from trials with a moderate or high risk of bias, provides valuable information about the efficacy and safety of movement interventions in patients affected by bone metastases.

## 4. Discussion

The aim of this systematic review was to describe and synthesize the available evidence regarding the impact of physical activity and exercise interventions on functional status and ADL performance in patients with bone metastases. We included eleven studies evaluating the effects of physical activity and movement-based interventions in advanced cancer patients affected by bone metastases, focusing on outcomes related to physical function and ADLs. Despite the heterogeneity of the included studies in terms of design, type of intervention, population, and assessment tools, the narrative synthesis highlighted consistent evidence supporting the functional benefits of such interventions in this population. Indeed, movement-based interventions, whether structured or adapted, have been consistently associated with improvements in physical function and independence in ADLs.

Our results are in line with those reported by Weller et al. [31] in their systematic review, in which physical exercise has been shown to be safe and potentially beneficial for functional capacity, quality of life and symptom control in patients with bone metastases, despite some methodological heterogeneity among the included studies. Instead, our review is partially in line with the study by Rogers-Shepp et al. [30] where some studies seem promising to improve physical function, but there is not enough evidence to understand whether this type of intervention improves quality of life.

The results of a recently published systematic review [29] show that exercise, when appropriately tailored to individual risk and supervised by experienced professionals, is generally safe and well tolerated even in this clinically complex population. Documented benefits include improvements in physical function (such as strength and endurance), reduced fatigue, increased ability to manage pain, and stability or improved quality of life. Efficacy was greater in personalized interventions that used bone risk assessment tools, such as Mirel’s score, and that included direct supervision. These results are confirmed by a narrative review [12] that provides a detailed overview of the clinical and practical considerations for the prescription of physical exercise in patients with bone metastases, which states how fundamental an assessment of bone risk, in particular through tools such as Mirel’s score and imaging, is to avoid pathological fractures. In fact, metastatic locations (e.g., vertebrae, proximal femur) require caution in the prescription of high-load or impact exercises. Exercise must be individualized, progressive and supervised, with particular attention to the patient’s symptoms (pain, fatigue, risk of falls).

In comparison with these previous reviews, this manuscript provides a complementary and more focused perspective. The review by Weller et al. [31] primarily investigated the safety and feasibility of exercise interventions in patients with bone metastases, with particular emphasis on muscle strength as a primary outcome. The review by Toohey et al. [29] instead examined the effects of physical activity in palliative care patients with advanced cancer, without specifically addressing bone metastases, and considered outcomes such as depression, pain, sleep, and anxiety. In contrast, this systematic review specifically aims to synthetize the studies with a specific focus on movement-related outcomes, functional autonomy and performance of activities of daily living, as endpoints for the role of exercise interventions in patients with bone metastases.

These findings highlight the importance of integrating individualized physical activity interventions into the supportive care of patients with bone metastases, underscoring their safety and potential to improve physical function and independence in daily living. The consistency of functional improvements observed across studies, despite variations in design and intervention types, suggests that exercise may represent a valuable therapeutic component even in clinically complex cancer populations. Evidence supports the integration of individualized and supervised exercise programs, particularly those guided by appropriate bone risk assessment tools, into multidisciplinary care pathways, with the aim of preserving function, promoting independence, and improving the overall care experience in advanced cancer settings. Within this framework, healthcare professionals play a crucial role in educating patients, tailoring safe exercise programs, supporting the maintenance of residual mobility, and ensuring that physical activity is effectively integrated into cancer care. Moreover, patients’ organizations can provide essential support by raising awareness, facilitating access to resources, and fostering a culture that values movement and exercise as an integral part of survivorship.

### 4.1. Implication for Practice and Future Research

The results of this systematic review highlight the value of a structured and personalized integrative physical activity intervention for the clinical management of patients with bone metastases. The recurrent functional improvement observed despite the heterogeneity of study designs and populations represents the best evidence of the possibility and therapeutic potential of a movement-based intervention, even in the medically compromised group. These interventions are able to maintain or even improve physical function and independence in daily activities, the fundamental parameters of quality of life and personal dignity in advanced oncological cases.

From a clinical point of view, the results of these studies confirm the inclusion of physical activity programs in the palliative and supportive management of patients with bone metastases. To ensure the safety and efficacy of use, the intervention should be followed by a careful assessment of skeletal stability, using tools such as Mirel’s score, and personalized according to the clinical status, symptoms and goals of the patient. Therefore, the management of these patients by a multidisciplinary team represents the best guarantee to mitigate risks and optimize results.

To minimize bias and improve the body of evidence, future research should use more rigorous designs, such as suitably powered RCTs with suitable control groups and assessor blinding. To evaluate whether functional benefits are sustainable over time, long-term monitoring is required. Findings will be more clinically relevant if there is a greater emphasis on patient-centered outcomes like independence, symptom burden, and perceived quality of life.

### 4.2. Strengths and Limitations

This systematic review has several strengths. It is, to our knowledge, the first to specifically summarize the evidence regarding the impact of movement-based interventions on physical function and ADLs in patients with bone metastases. By focusing on these functional outcomes, rather than solely on quality of life or general feasibility, this review fills a critical gap in the literature. However, some limitations must be acknowledged. The included studies were heterogeneous in terms of study design, sample size, intervention types, outcome measures, and assessment tools, which limited the ability to conduct a meta-analysis and directly compare results. Methodological quality was variable, with only two studies classified as low risk of bias, while most were moderate to high risk due to issues such as small sample size, lack of control groups, lack of blinding (due to the nature of the treatment) or incomplete follow-up (due to the poor prognosis of this type of patients). Indeed, the lack of long-term follow-up data in many studies makes it difficult to determine the sustainability of the observed functional benefits over time. A further limitation that should be acknowledged is the selection bias of the included studies. Most studies enrolled relatively small and highly selected samples, often restricted. As a result, the generalizability of these findings to the broader population of patients with bone metastases, many of whom present with poorer functional status or more advanced disease, could be limited.

Despite these limitations, the consistency of positive functional outcomes across different settings reinforces the potential value of physical activity interventions in improving independence and preserving function in patients with bone metastases.

## 5. Conclusions

This systematic review provides evidence that movement-based and physical activity interventions can play a meaningful role in the supportive care of patients with bone metastases. Although the included studies varied in design and clinical context, the findings consistently demonstrate improvements in physical function and autonomy in daily activities, core dimensions of quality of life and personal dignity in advanced cancer. While methodological limitations persist, the overall trends support the integration of personalized and supervised exercise programs into multidisciplinary care pathways. When guided by appropriate risk assessment and tailored to patient-specific conditions, these interventions can contribute to preserving function, reducing symptom burden, and enhancing overall well-being. Future research should aim to consolidate these findings through rigorous study designs, using uniform and reliable methods to assess outcomes, and long-term follow-up to better define the role of physical activity in this complex patient population.

## Figures and Tables

**Table 1:** Characteristics of the studies.

Author, Year-Country	Study Type	Population, Disease	Intervention	Outcomes (Tool)	Main Results	Conclusion
Born et al., 2010-Netherlands [47]	Case report	1 patient, breast cancer with bone metastases	“Life in Balance” Intervention	QoL—physical functioning (RAND-36)	Unchanged	Functional capacity improved to clinically meaningful levels. Despite some unchanged QoL subscales from disease progression, the intervention demonstrated gains in physical function
				Functional capacity (6MWT)	Improved significantly by 158 m	
Cormie et al., 2014-Australia [42]	Pilot study with a single-group longitudinal design	20 patients, prostate or breast cancer with bone metastases	Supervised resistance training combined with home-based aerobic exercise	Muscle strength- leg extension (1RM test)	Significant improvement after 3 months (+4%, *p* < 0.05)	Safe, acceptable, improved physical function and mobility, reduced fall and fracture risk, increased activity, enhanced QoL
				Aerobic capacity (400 m walk test)	Significant reduction in time (−3%, *p* < 0.05) post-intervention	
				Ambulation (6MWT)	Significant improvement at 3 months (−6% and −5%, *p* < 0.05); sustained at 6 months only for usual pace (−4%)	
				Mobility and balance confidence (TUG and ABC Scale)	Positive trends at 3 months (−4% in TUG time, +5% in balance confidence)	
				Leisure-time PA (GLTEQ)	Significant post-intervention increases in leisure score and weekly resistance training (*p* < 0.05), partially maintained at 6 months	
				Mild-intensity exercise (GLTEQ)	Positive trend at 3 months (*p* = 0.095), significant increase at 6 months	
				Physical functioning and Physical health composite (SF-36)	Positive trends at 3 months (+5% each; *p* = 0.095)	
Rief et al., 2014-Germany (a) [37]	Prospective, randomized, single-center, controlled pilot intervention study	60 patients, spinal bone metastases	Supervised isometric training with continued home practice	Mobility (Chair-stand, activity questionnaire)	Significantly improved in the intervention group (*p* < 0.001), while no change was seen in controls	Safe, effective, enhanced mobility, maintained improvements, no adverse effects
Rief et al., 2014-Germany (b) [38]	Randomized pilot study	60 patients, stable spinal bone metastases in the thoracic, lumbar, and sacral spinal segments	Supervised isometric training for spine/legs vs. passive therapy	QoL–Functional interference (EORTC QLQ–BM22)	Positive trend in the intervention group after 6 months (*p* = 0.081). ES = −0.56	Feasible, well tolerated, a significant reduction in daily life interference after 6 months, improved independence and mobility. Larger controlled studies are needed to confirm these results
				Interference with daily life (EORTC QLQ–FA13)	Significant reduction in the intervention group after 6 months (*p* = 0.006)	
Abe et al., 2016-Japan [44]	Observational study	25 patients, advanced cancer with spinal bone metastases and paraplegia	Horizontal movement system developed to facilitate patient transfer	Functional status (BI)	BI increased from 10.0 to 40.0 (*p* < 0.001). 92% of patients achieved transfer ability; 64% did so independently	Safe transfers, increased independence, improved functional mobility and ADL autonomy, without adverse effects
Galvão et al., 2017-Australia [40]	RCT	57 patients, prostate cancer with bone metastases	Modular Multimodal Exercise Program	Self-reported physical function (SF-36, physical functioning Subscale)	Significant increase in the exercise group vs. control (+3.2 points; *p* = 0.028)	Safe, well tolerated, improved perceived physical function and lower limb strength, no increase in pain, adaptable to metastatic site location
				Objective physical function (Timed Up-and-Go, 6 m usual and fast walk, 400 m walk test	Tests did not show significant change	
Yee et al., 2019-Australia [45]	Randomized Feasibility Trial	14 patients (29% bone-only metastases), metastatic breast cancer	Home-based exercise program or exercises in a local park vs. habitual physical activity	Physical functioning (EORTC QLQ-C30, physical functioning subscale)	CI 95% Glass’s delta = 1.71	Feasible, safe, high adherence to resistance training, poor adherence to walking, improved fatigue, physical function, and QoL
				PA objective (ACTi heart monitor PAEE)	Exercise group: 3143 ± 2362 kJ vs. Control group: 2714 ± 814 kJ, Glass’s delta = 0.59	
				PA self-reported (IPAQ)	Exercise group: 1709 ± 1785 vs. Control group: 1898 ± 2471, Glass’s delta = 0.10	
Groen et al., 2021-Netherlands [43]	Uncontrolled, single-arm, observational feasibility study	55 patients (75% with bone metastases), metastatic breast cancer	Customized physical therapy	Functional exercise capacity (6MWT)	Increased from 407 m to 481 m (+73.8 m)	Feasible, moderate improvements in walking and ADL, high goal attainment, patient satisfaction
				ADL Satisfaction (USER-P)	Increased from 59.1 to 65.3 points (+6.2; effect size 0.33)	
				ADL Restrictions (USER-P)	Slight improvement from 74.9 to 77.6 points (+2.7; effect size 0.16)	
				Physical functioning (EORTC QLQ–C30, physical functioning subscale)	Increased from 73.1 to 75.1 points (+1.9; effect size 0.11)	
Guinan et al., 2022-Ireland [46]	Observational cross-sectional study	58 patients, breast or prostate cancer with bone metastases	MVPA	PA (accelerometry-ACTi Graph-worn for 7 days)	About half of participants (48.3%) met PA guidelines (344 min/week MVPA). Those with fractures were significantly more sedentary and engaged in less light activity, while MVPA levels did not differ between groups	MVPA is associated with better pain control, improved physical function, and less interference in ADL. Fracture history was associated with greater sedentary time and lower light activity, highlighting the need for tailored exercise and education in this population
				Pain severity interference (BPI)	MVPA was inversely correlated with worst pain (r = −0.27, *p* = 0.04), average pain (r = −0.32, *p* = 0.015), current pain (r = −0.285, *p* = 0.03), and pain interference (r = −0.304, *p* = 0.02)	
				Physical functioning EORTC QLQ–C30, physical functioning subscale)	Significantly better in active participants (physical functioning subscale = 83.8 ± 16.7 vs. 67.3 ± 21.1, *p* = 0.002; r = 0.365, *p* = 0.005)	
				Functional interference (EORTC QLQ–BM22)	Improved in those meeting guidelines (80.7 vs. 64.6, *p* = 0.014)	
Pajares et al., 2022-Spain [41]	Prospective study	30 patients (22 with bone metastases), metastatic breast cancer	Supervised therapeutic exercise program	Functional capacity of the lower limbs (30-STS)	Increased from 14.50 to 19.61 repetitions	Feasible, safe, low adherence, improved in lower limb function and general mobility, slight decline in self-reported functionality
				Functional mobility (Lie-to-Sit Test)	Slight increase from 7.87 to 8.00 repetitions	
				Upper limb function (ULFI)	Decreased by 4.54 points	
				Lower limb function (LLFI)	Decreased by 3.28 points	
				PA level (IPAQ-SF)	Increased by 71 METs	
Hiensch et al., 2024-Germany, Netherlands, Poland, Spain, Sweden, and Australia [39]	RCT	357 patients (67% with bone metastases), metastatic breast cancer	Supervised exercise program	Self-reported PA (modified version of the GLTEQ)	Vigorous-intensity activity increased by +24 min/week (95% CI = 15–33), ES = 0.77; Resistance training increased by +38 min/week (95% CI = 27–49), ES = 1.49; Moderate activity no significant between-group difference; Light activity stable or slightly decreased	Safe, improve physical function, reduces sedentary behavior, and facilitates integration of exercise into supportive care
				Objective physical function (Fitbit Inspire HR)	Very active time: increased by +7 min/day (95% CI = 2–12), ES = 0.36; Sedentary time: decreased by −65 min/day (95% CI = −135 to −5), ES = 0.24 at 6 months	

Abbreviations: 30-STS—30-Second Sit-to-Stand Test; ULFI—Upper Limb Functional Index; LLFI—Lower Limb Functional Index; PA—Physical activity; IPAQ-SF—International Physical Activity Questionnaire—Short Form; METs—Metabolic Equivalents; EORTC QLQ–BM22—European Organisation for Research and Treatment of Cancer Quality of Life Questionnaire—Bone Metastases; EORTC QLQ–FA13—European Organisation for Research and Treatment of Cancer Quality of Life Questionnaire—Fatigue; EORTC QLQ–C30—European Organisation for Research and Treatment of Cancer Quality of Life Questionnaire—Core 30; 1RM Test—One Repetition Maximum Test; 6MWT—6-Minute Walk Test; TUG—Timed Up and Go Test; ABC Scale—Activities-specific Balance Confidence Scale; SF-36—36-Item Short Form Health Survey; RAND-36—RAND 36-Item Health Survey; BI—Barthel Index; ADL-Activities of Daily Living; USER-P—Utrecht Scale for Evaluation of Rehabilitation–Participation; IPAQ—International Physical Activity Questionnaire; PAEE—Physical Activity Energy Expenditure; GLTEQ—Godin Leisure-Time Exercise Questionnaire; BPI—Brief Pain Inventory; MVPA—Moderate-to-vigorous physical activity; ES—Effect Size; *p*—*p*-value; r—Pearson Correlation Coefficient; CI—Confidence Interval; RCT—Randomized Controlled Trial.

**Table 2:** Characteristics of exercise and movement-based interventions.

Study	Type of Intervention	Frequency	Duration per Session	Total Program Duration
Born et al., 2010 [47]	multimodal supervised group exercise program, including warm-up, circuit training (strength, mobility, coordination), individualized aerobic exercise (bike, treadmill, rowing), cool-down, occasional relaxation/breathing sessions	One session per week	90 min	9 weeks
Cormie et al., 2014 [42]	8 modularly selected exercises for the major muscle groups (2–4 sets, progressed from 12 to 8 repetitions maximum)	Two sessions per week	60 min	3-month and 6-month observation period
Rief et al., 2014 (a) [37]	isometric resistance training of the paravertebral (spinal) muscles, guided initially by a physiotherapist. Control group received respiratory therapy and “hot roll” treatment	One session per day (Monday–Friday) for 2 weeks; then 3 times per week for 12 weeks	30 min	2 weeks supervised + 12 weeks home-based (14 weeks total)
Rief et al., 2014 (b) [38]	guided isometric resistance training of the paravertebral muscles (intervention group), compared to passive physical therapy (breathing exercises, control group)	One session per day (Monday–Friday) for 2 weeks; then 3 times per week	30 min	2 weeks supervised + up to 6 months home-based
Abe et al., 2016 [44]	horizontal transfer method (using a transfer board and adjustable bed with safety apparatus) to move patients from bed to wheelchair	not standardized in number of sessions	not explicitly reported	intervention delivered during inpatient palliative care stay
Galvão et al., 2017 [40]	Supervised modular multimodal exercise program (resistance, aerobic, and flexibility training)	Three sessions per week	60 min	12 weeks (36 planned sessions; mean 32 attended)
Yee et al., 2019 [45]	Combined supervised and home-based program: brisk walking (10–15 min) + resistance training (30–40 min), individualized, moderate intensity	Two supervised sessions/week (16 total) + unsupervised walking on other days	40–55 min	8 weeks
Groen et al., 2021 [43]	Tailored, goal-directed physical therapy program (resistance, endurance, functional, relaxation exercises; supervised, home-based or blended with eHealth)	One/two sessions per week (as reported in the study)	standard physiotherapy session	12 weeks
Guinan et al., 2022 [46]	Habitual physical activity measured by accelerometer and questionnaires	Physical activity monitored over 7 consecutive days	Not reported (habitual activity, not structured sessions)	Not applicable (cross-sectional, 1-week monitoring)
Pajares et al., 2022 [41]	Individualized therapeutic exercise supervised by a physiotherapist (strength exercises and endurance with aerobic training)	Two sessions per week	60 min	12 weeks (22 sessions; completion ≥ 17 sessions)
Hiensch et al., 2024 [39]	Supervised, structured, and individualized exercise program including aerobic, resistance, balance, and flexibility training	Two sessions/week for 6 months; 1 supervised + 1 unsupervised session/week in months 7–9	60 min	9 months (median attendance 77%)

## Data Availability

All data in this systematic review are from published literature and are available through the original publications, which are cited in the article.

## Supplementary Materials

The following supporting information can be downloaded at: https://www.mdpi.com/article/10.3390/cancers17193266/s1, Supplementary File S1: Search strings; Supplementary File S2: Quality Assessment of Included Studies Using JBI Checklists.
