# Supplementary material for: Movement-Based Interventions in Patients Affected by Bone Metastases: Impact on Physical Function and Functional Autonomy—A Systematic Review
Source: Cancers (Basel). 2025 Oct 9;17(19):3266. doi: 10.3390/cancers17193266 (PMC12523373; doi:10.3390/cancers17193266)
Supplement: Supplementary file 1 [file cancers-17-03266-s001.zip › cancers-3857772-supplementary.pdf]

Supplementary file S1: Search strings

PubMed 24/03/2025

#1	"Neoplasm Metastasis"[Mesh] OR "neoplasm metastas*" OR "bone metastas*" OR "skeletal metastas*"	251,626
#2	mobility OR "Movement"[Mesh] OR "Locomotion"[Mesh] OR movement* OR locomotion* OR "Motor Activity"[Mesh] OR "Exercise"[Mesh] OR "motor activit*" OR exercis*	1,681,499
#3	"Functional Status" OR "Functional Dependence" OR "Functional Independence" OR "Activities of Daily Living"[Mesh] OR "Functional Status"[Mesh] OR "Activities Daily Living" OR "Daily Living Activit*" OR ADL OR "Chronic Limitation of Activity"	171,768
#4	#1 AND #2 AND #3	44

Scopus 24/03/2025

#1	(TITLE-ABS-KEY ("neoplasm metastas*" OR "bone metastas*" OR "skeletal metastas*")	162.606
#2	(TITLE-ABS-KEY ("motor activity" OR exercise OR movement OR locomotion OR mobility OR "motor activit*"OR exercis* OR movement* OR locomotion*))	3,371,605
#3	(TITLE-ABS-KEY ("activities of daily living" OR "functional status" OR "functional dependence" OR "functional independence" OR "daily living activit*" OR adl OR "chronic limitation of activity"))	204.681
#4	#1 AND #2 AND #3	82

Cinahl 24/03/2025

#1	(MH "Bone Metastases" OR MH "Neoplasm Metastasis") OR ("neoplasm metastas*") OR ("bone metastas*") OR ("skeletal metastas*")	50.720
#2	(mobility) OR (MH "Movement") OR (MH "Locomotion") OR (movement*) OR (locomotion*) OR (MH "Motor Activity") OR (MH "Exercise+") OR ("motor activit*") OR (exercis*)	368.489
#3	MH "Functional Status" OR "Functional Dependence" OR "Functional Independence" OR MH "Activities of Daily Living" OR "Activities Daily Living" OR "Daily Living Activit*" AND "Chronic Limitation of Activity" OR ADL	77.299
#4	#1 AND #2 AND #3	52

Web of science 24/03/2025

#1	(ALL=("neoplasm metastas*") OR ALL=("bone metastas*") OR ALL=("skeletal metastas*"))	31.581
#2	(ALL=("motor activity") OR ALL=("exercise") OR ALL=("movement") OR ALL=("locomotion") OR ALL=("mobility") OR ALL=("motor activit*") OR ALL=("exercis*") OR ALL=("movement*") OR ALL=("locomotion*"))	2.405.167
#3	(ALL=("activities of daily living") OR ALL=("functional status") OR ALL=("functional dependence") OR ALL=("functional independence") OR ALL=("activities daily living") OR ALL=("daily living activit*") OR ALL=("ADL") OR ALL=("chronic limitation of activity"))	98.732
#4	#1 AND #2 AND #3	24

Embase 20/03/2025

#1	('metastasis' OR 'bone metastasis' OR 'musculoskeletal metastasis')	1.061.886
#2	('mobilization' OR 'mobility' OR 'movement (physiology)' OR 'locomotion' OR 'motor activity' OR 'exercise')	2.296.164
#3	('functional dependence' OR 'functional dependency' OR 'functional independence' OR 'daily life activity' OR 'functional status' OR 'activity of daily living assessment')	292.321
#4	#1 AND #2 AND #3	484

Supplementary file S2: Quality Assessment of Included Studies Using JBI Checklists

		Rief et al.2014	Rief et al.2014	Galvão et al. 2017	Hiensch et al. 2024	Yee et al. 2019
**1**	Was true randomization used for assignment of participants to treatment groups?	Yes	Yes	Yes	Yes	Yes
**2**	Was allocation to treatment groups concealed?	Unclear	Yes	Yes	Unclear	Yes
**3**	Were treatment groups similar at the baseline?	Yes	Yes	Yes	Yes	No
**4**	Were participants blind to treatment assignment?	N.A	N.A	N.A	N.A	N.A
**5**	Were those delivering the treatment blind to treatment assignment?	N.A	N.A	N.A	N.A	N.A
**6**	Were treatment groups treated identically other than the intervention of interest?	Yes	Yes	Yes	Yes	No
**7**	Were outcome assessors blind to treatment assignment?	Unclear	Unclear	Unclear	Unclear	No
**8**	Were outcomes measured in the same way for treatment groups?	Yes	Yes	Yes	Yes	Yes
**9**	Were outcomes measured in a reliable way	Yes	Unclear	Yes	Yes	Yes
**10**	Was follow up complete and if not, were differences between groups in terms of their follow up adequately described and analysed?	Yes	Yes	No	Yes	No
**11**	Were participants analysed in the groups to which they were randomized?	No	No	No	Yes	No
**12**	Was appropriate statistical analysis used?	Yes	Yes	Yes	Yes	Yes
**13**	Was the trial design appropriate and any deviations from the standard RCT design (individual randomization, parallel groups) accounted for in the conduct and analysis of the trial?	Yes	Yes	Yes	Yes	Yes

		Moderate	Moderate	Moderate	Moderate	High

		**Pajares et al. 2021**	**Cormie et al.2014**	**Groen et al. 2021**	**Abe et al. 2016**
**1**	Is it clear in the study what is the "cause" and what is the "effect" (i.e. there is no confusion about which variable comes first)?	Yes	Yes	Yes	Yes
**2**	Was there a control group?	No	No	No	No
**3**	Were participants included in any comparisons similar?	N/A	N/A	N/A	N/A
**4**	Were the participants included in any comparisons receiving similar treatment/care, other than the exposure or intervention of interest?	N/A	N/A	N/A	N/A
**5**	Were there multiple measurements of the outcome, both pre and post the intervention/exposure?	Yes	Yes	Yes	Yes
**6**	Were the outcomes of participants included in any comparisons measured in the same way?	N/A	N/A	N/A	Yes
**7**	Were outcomes measured in a reliable way?	Yes	Unclear	Yes	Unclear
**8**	Was follow-up complete and if not, were differences between groups in terms of their follow-up adequately described and analyzed?	Yes	Yes	Yes	Yes
**9**	Was appropriate statistical analysis used?	Yes	Yes	Yes	Yes
		High	High	High	High

		Born et al. 2010
**1**	Were patient's demographic characteristics clearly described?	Yes
**2**	Was the patient's history clearly described and presented as a timeline?	Yes
**3**	Was the current clinical condition of the patient on presentation clearly described?	Yes
**4**	Were diagnostic tests or assessment methods and the results clearly described?	Yes
**5**	Was the intervention(s) or treatment procedure(s) clearly described?	Yes
**6**	Was the post-intervention clinical condition clearly described?	Yes
**7**	Were adverse events (harms) or unanticipated events identified and described?	Yes
**8**	Does the case report provide takeaway lessons?	Yes
		Low

		**Guinan et al. 2022**
**1**	Were the criteria for inclusion in the sample clearly defined?	Yes
**2**	Were the study subjects and the setting described in detail?	Yes
**3**	Was the exposure measured in a valid and reliable way?	Yes
**4**	Were objective, standard criteria used for measurement of the condition?	Yes
**5**	Were confounding factors identified?	Yes
**6**	Were strategies to deal with confounding factors stated?	No
**7**	Were the outcomes measured in a valid and reliable way?	Yes
**8**	Was appropriate statistical analysis used?	Yes
		Low
